# Prevalence of Musculoskeletal Disorders among General and Technical Secondary School Students in Egypt

**DOI:** 10.3390/ijerph20021465

**Published:** 2023-01-13

**Authors:** Doaa Tammam Atia, Nader Ibrahim Elsayed, Asmaa Foad Abdelmonem, Sally Mohamed Sae’d Mahmoud, Marwa Mahmoud Mahfouz Mahmoud, Kamal Eldin S. Mohamed, Khalid Taha Yassin Turky, Usama M. Rashad, Amel E. Abdel Karim

**Affiliations:** 1 Faculty of Physical Therapy, Heliopolis University, Cairo 11785, Egypt; 2 National Institute of Neuromotor System, General Organization for Teaching Hospitals and Institutes, Cairo 11697, Egypt; 3 Faculty of Physical Therapy, Alhayah University, Cairo 11835, Egypt; 4 Faculty of Physical Therapy, Cairo University, Cairo 12613, Egypt; 5 Faculty of Physical Therapy, Deraya University, Minia 61754, Egypt; 6 Faculty of Physical Therapy, Galala University, Suez 43111, Egypt; 7 College of Physical Therapy, Misr University for Science and Technology, 6th of October City 12585, Egypt

**Keywords:** musculoskeletal disorders, technical schools, general schools, RULA, NMQ

## Abstract

(1) Background: Children spend a lot of time within schools. The school setting generally has many ergonomic hazards and reinforced behavior patterns which put children at greater risk of environmental hazards than adults during their critical developmental stages. (2) Objective: The aim of the current study was to investigate the prevalence of musculoskeletal disorders (MSDs) and detect spinal deformities amongst general and technical secondary school students. (3) Methods: A total of 418 students from the second grade of secondary school in Shaquira governorate, Egypt participated in this cross-sectional study. Each student in the study was screened via Nordic Musculoskeletal Questionnaire (NMQ) and had their upper limb posture measured via RULA (Rapid Upper Limb assessment), and the deviation in their thoracic curve was measured using a scoliometer. (4) Results: There was a prevalence of MSDs amongst students as there were 69.7% of general school students and 83.8% of the technical school students suffering from MSDs with a statistically significant difference between both technical and general school students in RULA score and musculoskeletal complaints, whereas there were non-statistical differences in the scoliometer scale in both general and technical education students. (5) Conclusions: Musculoskeletal problems are prevalent among Egyptian secondary school students, with higher prevalence between technical school students. Therefore, preventive measures and strategies are recommended to overcome the future complications of these musculoskeletal disorders.

## 1. Introduction

Musculoskeletal disorders (MSDs) are permanent injuries or pain in the body, including in muscles, joints, tendons, ligaments, nerves, and bones. MSDs have a high prevalence worldwide as a result of major occupational injuries in both developed and developing countries [1]. Adolescents complaining of musculoskeletal pain are more likely to develop chronic musculoskeletal pain in adulthood [2].

Many risk factors are frequently correlated with MSDs, including physical, psychological, social, and biomechanical hazards such as repeated motion, continuous forceful exertion, faulty postures, and vibration, all of which have harmful effects on the musculoskeletal system. These negative ergonomic factors, in addition awkward postures and working long hours without rest and in poor working conditions, are considered the main causes for MSDs [3,4]. More than 150 disorders and syndromes have been determined to result in decreased mobility and function and lowered quality of life. These MSDs were determined to be the leading cause for disability because of the recurrency of musculoskeletal disorders from adolescence into adulthood without appropriate treatment [5,6].

School students are susceptible to developing musculoskeletal pain because of their progressive growth and development. MSDs in school students vary in severity from light, transient, to severe disorders that limit physical activity, impair health, and affect quality of life [7].

Inappropriate postures and a lack of time for athletic activities can cause MSDs to start early in childhood [8]. The likelihood of recurrent musculoskeletal pain in adults increases if it is ignored in childhood or adolescence. Thus, high prevalence of MSDs among children raises the issue of young workers coming into the workplace with pre-existing musculoskeletal problems which could be exacerbated by work. So, by formulating MSDs prevention strategies targeting children and adolescents, adult MSDs prevalence may be reduced and the onset of a cycle of recurring episodes may be avoided [9,10,11].

The onset of MSDs occurs in adolescence, so it is important to investigate this disorder in this stage of life, or even earlier, to determine the initial onset, thus aiding in developing effective treatments and strategies for primary prevention [12].

Students spend at least 5 h a day in traditional schools. Previous studies revealed the prevalence of MSDs to vary between 10 and 67% of students. MSDs have an adverse effect on the daily activities and increase the rate of absenteeism from school [13].

Long periods of time spent in the same posture have serious negative effects on students’ health, including an increase in musculoskeletal complaints, discomfort at school, and fatigue during the day. The tolerance for repeating the same effort may be decreased by continuous and excessive exertion of force. The risk of MSDs increases when task requirements exceed the students’ abilities [14].

The aim of the present study is to provide adequate knowledge of prevalence of MSDs among secondary school students in either general or technical schools, which may help in reducing the occurrence of MSDs by establishing prevention strategies in the future.

It was hypothesised that there is no significant difference in NMQ, RULA score nor scoliometer reading between grades.

## 2. Materials and Methods

### 2.1. Study Design, Venue, and Time

In this cross-sectional study, both subjects and examiners were blinded to the study’s hypothesis in order to avoid false reported responses by either over/underestimation of symptoms, as the measurement was conducted via a self-reported questionnaire. The study was conducted in four secondary schools in Sharquia governorate, Egypt via semi-structured interviews that were conducted with the students. The study started in October 2021 and ended in March 2022 and was carried out through scheduled visits to the school arranged with the school principal. The study used cluster sampling via choosing representative schools for both general and technical schools.

### 2.2. Study Population

**Figure 1:**
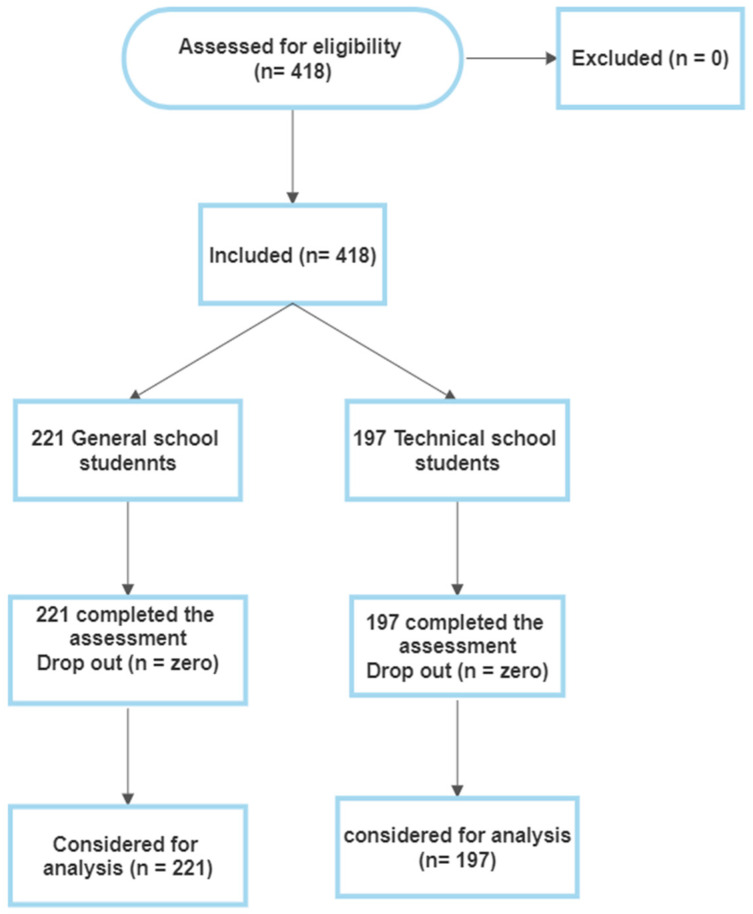
The flowchart of the participants.

In total, 418 students from the second grade of secondary school in Sharquia governorate, Egypt of both sexes participated; 221 general school students and 197 technical school students with ages ranging from 15 to 17 years old contributed to the current study. Figure 1 illustrates the flowchart of the participants of the study. This study was limited to students from the second grade of general and technical secondary schools in Sharquia governorate, Egypt with no congenital anomalies, injuries, or mental disabilities.

### 2.3. Measurements Tools

Musculoskeletal symptoms were investigated using a self-administered questionnaire with questions addressing personal and educational factors. The baseline questionnaire is constructed from the Nordic Musculoskeletal Questionnaire (NMQ) via direct interviews with the students [15]. The Arabic version is validated and has a Cronbach’s alpha (0.87) [16]. Upper limb posture was assessed using RULA (Rapid Upper Limb assessment). A single page worksheet was used to evaluate required body posture, force, and repetition. Based on the evaluations, scores were entered for each body region in section A for the arm and wrist, and section B for the neck and trunk. It was performed in confined spaces with the tested students without disturbance to the rest of the students [17]. A scoliometer was used to assess the lateral curvature of the thoracic region. Both students and their school bags were weighed on a digital scale.

### 2.4. Study Procedure

Students were presented with an introduction to MSDs and their effects in later adult stages. The presentation was conducted by the team of the researchers to inform students that detecting the presence of musculoskeletal problems and disorders could help in the prevention of more severe complications later. Before the beginning of the study, the entire procedure of the study, the questionnaire, the scale and scoliometer measurements were explained to the students and their parents in detail, either in a presentation form at the school or by sending out an illustrative brochure with the consent form.

### 2.5. Ethics

The procedures followed were agreed upon with the authorization of the Institutional Ethical Committee, and informed written consent was obtained. The signed informed consent for each subject was maintained by the investigator in strict confidence. participation acceptance was obtained from parents. Ethical approval: N. HU.REC.H-1-2020 Heliopolis university.

### 2.6. Data Analysis

The collected data were coded, tabulated, and statistically analyzed using IBM SPSS statistics (Statistical Package for Social Sciences) software version 28.0, IBM Corp., Chicago, USA, 2021. Quantitative data were tested for normality using the Kolmogorov–Smirnov test; if normally distributed, the data were then described as mean ± SD (standard deviation) as well as the minimum and maximum of the range and 95% confidence interval, then students’ age, BMI, RULA and scoliometer measurements were compared using an ANOVA test. Qualitative data were described as numbers and percentages, as well as 95% confidence intervals and compared using Chi-square test and Fisher’s Exact test for variables with small, expected numbers. Post hoc Bonferroni test was used to determine homogenous groups between the students of technical departments and general school. The level of significance of difference was taken at *p*-value ≤ 0.050 and non-significant otherwise.

## 3. Results

### 3.1. Demographic Characteristic of the Participant Students

**Figure 2:**
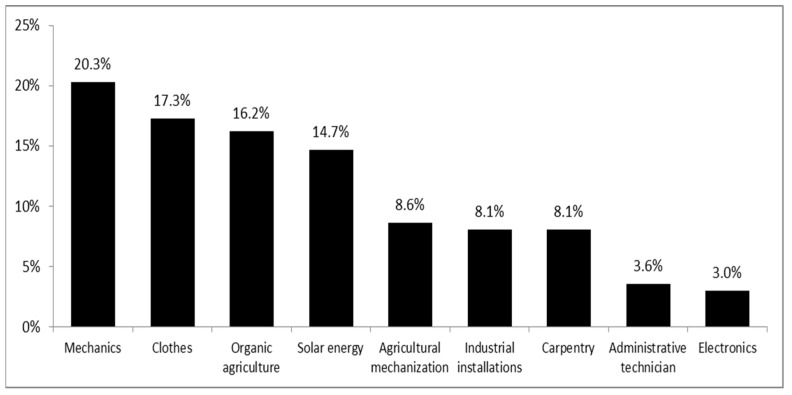
Technical specialties.

In total, 418 students (221 of general school students and 197 of technical school students) participated in the study. Among the technical schools’ students, 40 studied mechanics, 34 clothing, 32 organics, 29 solar energy, 17 agricultural mechanization, 16 industrial installations, 16 carpentry, 7 were in an administrative technician program, and 6 studied electronics. Their age ranged from 14 to 17 years old. There were no statistically significant differences corresponding to education regarding age, sex, and exercise between both groups, while BMI was statistically significantly lower in general education students than in those in technical education, as shown in Figure 2 and Table 1.

### 3.2. RULA Score and Scoliometer Scale among the Studied Groups

The RULA scale was statistically significantly the highest in electronics students, followed by industrial installations students, and was lowest in general school students followed by administrative technicians. Overall, the RULA scale was statistically significantly lower in students in general education than in those in technical education. On the other hand, there was non-significant difference in the scoliometer scale measurements between both general and technical school students, as shown in Table 2.

### 3.3. MSDs Complaints among the Studied Groups

#### 3.3.1. Musculoskeletal Complaints

Table 3 shows that musculoskeletal complaints were statistically significantly most frequent in electronics students followed by those in agricultural mechanization and were least frequent in general education students followed by those in solar energy. Overall, musculoskeletal complaints were statistically significantly less frequent in the general education group than in technical education.

#### 3.3.2. Neck and Back Complaints

Table 4 shows that neck complaints were statistically significantly most frequent in electronics students followed by administrative technicians and were least frequent in solar energy students followed by mechanics students. Overall, neck complaints were statistically significantly less frequent in students in general than in those in technical education. Upper back complaints were statistically significantly most frequent in electronics students followed by those in solar energy and were least frequent in organic agriculture students followed by those in general education. Lower back complaints were statistically significantly most frequent in industrial installations students followed by those in agricultural mechanization and were least frequent in general education students followed by those in mechanics. Overall, both upper back and lower back complaints were statistically significantly less frequent in the general education group than in technical education.

#### 3.3.3. Upper Limb Complaints

Table 5 shows that shoulder complaints were statistically significantly most frequent in agricultural mechanization students followed by those in electronics and were least frequent in solar energy students followed by administrative technicians. Overall, shoulder complaints were statistically non-significantly less frequent in general education students than in those in technical education. Elbow complaints were statistically significantly most frequent in electronics students followed by those in carpentry and were least frequent in general education students followed by those in agricultural mechanization. Wrist complaints were statistically significantly most frequent in organic agriculture students followed by those in carpentry and electronics, and were least frequent in mechanics students followed by those in industrial installations. Overall, both elbow and wrist complaints were statistically significantly less frequent in the general education group than in technical education.

#### 3.3.4. Lower Limb Complaints

Table 6 shows that hip complaints were statistically significantly most frequent in electronics students followed by those in solar energy and were least frequent in clothes students followed by the general school student group. Knee complaints were statistically significantly most frequent in electronics students followed by those in organic agriculture and were least frequent in agricultural mechanization students followed by those in industrial installations. Ankle complaints were statistically significantly most frequent in electronics students followed by those in mechanics and were least frequent in general school students followed by administrative technicians. Overall, both hip and ankle complaints were statistically significantly less frequent in the general education group than in technical education, while there was non-significant difference between both groups in knee complaints.

## 4. Discussion

This study was conducted to determine the prevalence of musculoskeletal disorders among general and technical secondary school students. No previous data about the MSDs amongst the adolescents in technical schools whose study days are totally different than those in the general education system were available. While general school students spend their school days sitting at desks, the technical school students apply and practice their courses according to their departments.

### 4.1. Risk Factors for the Prevalence of MSDs

The current study showed the prevalence of MSDs among secondary school students in different body regions. Previous studies have also shown that the prevalence of MSDs increases with increasing age and is most common among the adolescent age group compared to those of elementary and preparatory school [18,19]. Adolescents may have increased prevalence of MSDs due to increased activities and stresses as they become older. The organization of health services, the environment, cultural differences, or other unidentified factors may also contribute. However, the precise cause of this development remains unknown [20,21]. This agrees with the study by Sushmitha et al. [22] that determined a notable prevalence of MSDs among secondary school students due to the related risk factors including school bag weight, anthropometric measures, and other causes related to furniture mismatch. On the other hand, Ghazilla et al. [23] reported that prolonged periods spent in maintaining faulty postures could result in various musculoskeletal illness and postural deviations. Thus, for the best health and safety outcomes, the authors stated that employees in the workplace should change body positions between sitting, standing, and moving about.

There is a variety of risk factors that can lead to MSDs in children and adolescents. The most common risk factors of MSDs in general schools are heavy backpacks and inappropriate school furniture. A study by Murphy et al. [24] revealed that characteristics of school furniture have the highest prevalence of relationship with pain. Traditional workstations in schools are unsuitable for schoolchildren. Children must adopt flexed or static postures for prolonged periods, increasing muscular fatigue in both the neck and back.

The current study revealed that there is a highly significant difference in RULA scores between general and technical school students, with the highest score being in electronics students followed by industrial installation students and is the lowest in general school schools as there is continuous use of the upper limb in awkward positions. This finding was in support of Habibi et al. [25], who determined that computer users, according to the type and nature of their jobs, are at a greater risk of MSDs. Based on the RULA scores, more than 30% of the study sample was at a greater risk of developing MSDs as a result of working for a long time in semi-static work positions, inadequate rest, and awkward postures while working with computers. In addition, Gheysvandi et al. [26] determined a significant relationship between bending and rotating the neck and higher RULA scores and musculoskeletal neck pain among school-age children and adolescents due to their poor postures and prolonged sitting among school students aged 7 to 12 years old from Hamadan city, Iran.

The current study revealed a higher prevalence of musculoskeletal disorders (MSDs) among the students at technical schools than in those in general schools; students at general schools also suffered from musculoskeletal pain but with a non-significant statistical value compared with the students in the technical schools. The difference in the school environment and the risk factors which include mechanical workload such as awkward postures, manual material handling, prolonged sitting and standing, repetitive movements, and excessive load may be the main cause for musculoskeletal problems in students in technical schools. This was supported by previous studies among newly employed subjects which determined the direct association between the mechanical workload such as lifting, pulling, squatting, and prolonged raising of hands above shoulder height and multisite musculoskeletal pain [27,28,29]. Other studies revealed that physical demands at work, including often identified occupational risk factors such as increased muscle loads; working and lifting in stooping, restrictive, or twisted postures; repetitive movements; and working with lifted arms play a significant role in the development of MSDs [30,31].

### 4.2. Sites of MSDs Pain

The current study revealed that spine complaints, either in the form of neck or back pain, were statistically significantly less frequent in general education students than in those in technical education. Neck complaints were statistically significantly most frequent in electronics students followed by administrative technicians. Upper back complaints were statistically significantly most frequent in electronics students followed by those in solar energy. Lower back complaints were statistically significantly most frequent in industrial installations students followed by those in agricultural mechanization. Overall, both upper back and lower back complaints were statistically significantly less frequent in the general education group than in technical education. These findings are in agreement with previous study by Sun et al. [32], who confirmed findings from previous studies about the causal relationship between neck MSDs and underlying risk factors such as repetitive movements, especially in combination with forceful exertions, awkward working postures, poor workstation design, and high exposure to vibration. Most research on neck and upper limb symptoms focused on work-related physical exposure. Reviews of previous studies for physical risk factors for neck pain reported moderate evidence for a correlation between both forceful arm exertions and heavy lifting and neck–shoulder pain. A linkage between neck–shoulder disorders and occupational risk factors, such as repetition of movements, physical workload, and prolonged static efforts, was also observed [33,34].

On the other hand, Peek-Asa et al. [35], in his study on approximately 50,000 material handling workers employed in Home Depot stores in California, USA, determined that there are biomechanical risk factors identified for the development of low back MSDs, mainly heavy physical work and lifting and awkward static and dynamic working postures. Incorrect posture and prolonged sedentary positions are commonly associated with lower back pain in children and adolescents and negative health outcomes [36]. Although mild back pain is most common across all age groups, it seems remarkable that, as people age, reports of intense pain increase in frequency. In the age range of 17–19 compared to the age group of 10–13, strong pain happens twice as frequently [37]. Back pain was reported by 74.4% of participants in an American study by Sheir-Neiss et al. that included children aged 12 to 18 [38]. Nearly 60% of adolescents from Nigeria reported having back pain, according to studies by Ayanniyi et al. [39].

The prevalence of shoulder and wrist pain amongst the students may be a direct result of biomechanical risk factors including heavy physical work, repetitive work, awkward static and dynamic working postures, and prolonged electronic work. This was corroborated by a previous study by Andersen et al. [40], who studied 5604 workers of the general working population in western Denmark from 39 different workplaces (19 in the service sector and 20 in different kinds of industries). The study determined that subjects with high levels of repetitive work had higher liability to an increased risk of arm pain while lifting at or above shoulder level, and the risk remained significant for neck/shoulder pain.

In the current study, the prevalence of MSDs with a significant effect on ankle joint may be due to heavy physical work with prolonged kneeling or squatting positions, and prolonged time of standing; lifting heavy loads may also explain the significant prevalence of lower limb MSDs. This consistent with a previous study of 859 newly employed workers from 12 diverse occupational settings in England, which revealed that standing for most of the time without freedom to sit while handling heavy loads, performing repetitive hand and arm movements, being subjected to physical violence at work and high job strain are risk factors for developing lower limb pain [41,42].

## 5. Conclusions

The current study revealed that MSDs are prevalent among secondary school children, especially those in the technical schools who suffer from multi-site pain due to predisposing risk factors. Increased student awareness level and prevention measures should be added to overcome the leading risk factors of developing MSDs.

## Figures and Tables

**Table 1:** Demographic characteristics of the participating students.

Demographic Data		General	Technical	*p*-Value
Age		15.9 ± 0.7	15.9 ± 0.8	0.427
Sex	Male	109 (49.3%)	101 (51.3%)	0.691
	Female	112 (50.7%)	96 (48.7%)	
BMI		22.3 ± 3.7	23.3 ± 4.3	0.008 *
Exercises		128 (57.9%)	109 (55.3%)	0.594

* Significant: *p*-value < 0.05.

**Table 2:** RULA and Scoliometer Scale among the studied groups.

Detailed Education Types							
Education	Total	RULA			Scoliometer		
		Mean ± SD	95% CI	HG	Mean ± SD	95% CI	HG
General	221	5.1 ± 1.3	4.9–5.2	a	2.6 ± 2.8	2.3–3.0	a
Mechanics	40	6.0 ± 1.2	5.6–6.3	a, b	2.1 ± 2.2	1.4–2.8	a
Clothes	34	5.6 ± 1.3	5.1–6.0	a	2.1 ± 2.1	1.4–2.9	a
Organic agriculture	32	5.9 ± 1.4	5.4–6.4	a, b	2.3 ± 2.9	1.2–3.3	a
Solar energy	29	6.2 ± 1.3	5.7–6.7	a, b	3.3 ± 3.1	2.2–4.5	a
Agricultural mechanization	17	6.1 ± 1.2	5.5–6.7	a, b	1.1 ± 2.2	0.0–2.3	a
Industrial installations	16	6.4 ± 1.0	5.9–7.0	a, b	2.7 ± 2.3	1.4–3.9	a
Carpentry	16	6.1 ± 1.0	5.6–6.7	a, b	3.0 ± 2.3	1.8–4.2	a
Administrative technician	7	5.1 ± 0.7	4.5–5.8	a	1.9 ± 2.3	−0.3–4.0	a
Electronics	6	7.0 ± 0.0	7.0–7.0	b	2.0 ± 2.2	−0.3–4.3	a
^ *p*-value	<0.001 *				0.255		
**Main Education Types**							
General	221	5.1 ± 1.3	4.9–5.2		2.6 ± 2.8	2.3–3.0	
Technical	197	6.0 ± 1.2	2.0–7.0		2.3 ± 2.5	0.0–11.0	
# *p*-value	<0.001 *				0.284		

^ ANOVA test. # Independent *t*-test. * Significant: *p*-value < 0.05. CI: Confidence interval. HG: Homogenous groups based on post hoc Bonferroni test, homogenous groups had the same symbol “a, b”.

**Table 3:** Musculoskeletal complaints among the studied groups.

Education	Total	MSDs	95% CI	HG
**Detailed education types**				
Traditional	221	154 (69.7%)	63.4–75.5%	a
Mechanics	40	32 (80.0%)	65.8–90.1%	a
Clothes	34	27 (79.4%)	63.8–90.3%	a
Organic agriculture	32	28 (87.5%)	73.0–95.6%	a
Solar energy	29	23 (79.3%)	62.2–90.9%	a
Agricultural mechanization	17	15 (88.2%)	67.3–97.5%	a
Industrial installations	16	14 (87.5%)	65.6–97.3%	a
Carpentry	16	14 (87.5%)	65.6–97.3%	a
Administrative technician	7	6 (85.7%)	49.9–98.4%	a
Electronics	6	6 (100.0%)	Not applicable	a
# *p*-value	0.123			
**Main education types**				
Traditional	221	154 (69.7%)	63.4–75.5%	
Technical	197	165 (83.8%)	78.1–88.4%	
^ *p*-value	<0.001 *			

^ Chi square test. # Fisher’s Exact test. * Significant: *p*-value < 0.05. CI: Confidence interval. HG: Homogenous groups based on post hoc Bonferroni test, homogenous groups had the same symbol “a”.

**Table 4:** Neck and back complaints among the studied groups.

Education	Neck Pain			Upper Back Pain			Lower Back Pain		
	Mean	95% CI	HG	Mean	95% CI	HG	Mean	95% CI	HG
General	43 (19.5%)	14.7–25.1%	a, b	47 (21.3%)	16.3–27.0%	a	52 (23.5%)	18.3–29.4%	a
Mechanics	6 (15.0%)	6.5–28.3%	a, b	14 (35.0%)	21.7–50.4%	a, b	10 (25.0%)	13.6–39.8%	a, b
Clothes	14 (41.2%)	25.9–57.9%	a, b	12 (35.3%)	20.9–52.0%	a, b	13 (38.2%)	23.4–55.0%	a, b
Organic agriculture	10 (31.3%)	17.3–48.4%	a, b	6 (18.8%)	8.2–34.6%	a	9 (28.1%)	14.9–45.1%	a, b
Solar energy	4 (13.8%)	4.8–29.5%	a	14 (48.3%)	31.0–65.9%	a, b	14 (48.3%)	31.0–65.9%	a, b
Agricultural mechanization	7 (41.2%)	20.7–64.4%	a, b	5 (29.4%)	12.2–53.0%	a, b	9 (52.9%)	30.3–74.6%	a, b
Industrial installations	6 (37.5%)	17.4–61.7%	a, b	2 (12.5%)	2.7–34.4%	a	11 (68.8%)	44.4–86.9%	b
Carpentry	6 (37.5%)	17.4–61.7%	a, b	7 (43.8%)	22.2–67.4%	a, b	7 (43.8%)	22.2–67.4%	a, b
Administrative technician	3 (42.9%)	13.9–76.5%	a, b	1 (14.3%)	1.6–50.1%	a, b	3 (42.9%)	13.9–76.5%	a, b
Electronics	3 (50.0%)	16.7–83.3%	b	6 (100.0%)	Not applicable	b	3 (50.0%)	16.7–83.3%	a, b
# *p*-value	0.010 *			<0.001 *			<0.001 *		
General total	43 (19.5%)	14.7–25.1%		47 (21.3%)	16.3–27.0%		52 (23.5%)	18.3–29.4%	
Technical total	59 (29.9%)	23.9–36.6%		67 (34.0%)	27.7–40.8%		79 (40.1%)	33.4–47.0%	
^ *p*-value	0.013 *			0.003 *			<0.001 *		

^ Chi square test. # Fisher’s Exact test. * Significant: *p*-value < 0.05. CI: Confidence interval. HG: Homogenous groups based on post hoc Bonferroni test, homogenous groups had the same symbol “a, b”.

**Table 5:** Upper limb complaints among the studied groups.

Education	Shoulder Pain			Elbow Pain			Wrist Pain		
	Mean	95% CI	HG	Mean	95% CI	HG	Mean	95% CI	HG
General	42 (19.0%)	14.3–24.6%	a, b	4 (1.8%)	0.6–4.2%	a	49 (22.2%)	17.1–28.0%	a, b
Mechanics	10 (25.0%)	13.6–39.8%	a, b, c	4 (10.0%)	3.5–22.0%	a, b	4 (10.0%)	3.5–22.0%	b
Clothes	8 (23.5%)	11.8–39.5%	a, b, c	4 (11.8%)	4.1–25.6%	a, b	14 (41.2%)	25.9–57.9%	a, b, c
Organic agriculture	8 (25.0%)	12.6–41.7%	a, b, c	4 (12.5%)	4.4–27.0%	a, b	17 (53.1%)	36.2–69.5%	c
Solar energy	0 (0.0%)	Not applicable	b	4 (13.8%)	4.8–29.5%	b	8 (27.6%)	14.0–45.4%	a, b, c
Agricultural mechanization	9 (52.9%)	30.3–74.6%	c	1 (5.9%)	0.6–24.4%	a, b	5 (29.4%)	12.2–53.0%	a, b, c
Industrial installations	4 (25.0%)	9.1–49.1%	a, b, c	1 (6.3%)	0.7–25.7%	a, b	3 (18.8%)	5.6–42.1%	a, b, c
Carpentry	4 (25.0%)	9.1–49.1%	a, b, c	4 (25.0%)	9.1–49.1%	b	8 (50.0%)	27.2–72.8%	a, c
Administrative technician	0 (0.0%)	Not applicable	a, b, c	0 (0.0%)	Not applicable	a, b	2 (28.6%)	6.5–64.8%	a, b, c
Electronics	3 (50.0%)	16.7–83.3%	a, c	3 (50.0%)	16.7–83.3%	b	3 (50.0%)	16.7–83.3%	a, b, c
# *p*-value	0.003 *			<0.001 *			<0.001 *		
General total	42 (19.0%)	14.3–24.6%		4 (1.8%)	0.6–4.2%		49 (22.2%)	17.1–28.0%	
Technical total	46 (23.4%)	17.9–29.6%		25 (12.7%)	8.6–17.9%		64 (32.5%)	26.2–39.2%	
^ *p*-value	0.227			<0.001 *			0.018 *		

^ Chi square test. # Fisher’s Exact test. * Significant: *p*-value < 0.05. CI: Confidence interval. HG: Homogenous groups based on post hoc Bonferroni test, homogenous groups had the same symbol “a, b, c”.

**Table 6:** Lower limb complaints among the studied groups.

Education	Hip Pain			Knee Pain			Ankle Pain		
	Mean	95% CI	HG	Mean	95% CI	HG	Mean	95% CI	HG
General	3 (1.4%)	0.4–3.6%	a	55 (24.9%)	19.5–30.9%	a	18 (8.1%)	5.1–12.3%	a
Mechanics	4 (10.0%)	3.5–22.0%	a, b, c	12 (30.0%)	17.6–45.2%	a	12 (30.0%)	17.6–45.2%	b
Clothes	0 (0.0%)	Not applicable	a, c	9 (26.5%)	14.0–42.8%	a	8 (23.5%)	11.8–39.5%	a, b
Organic agriculture	6 (18.8%)	8.2–34.6%	b, c	13 (40.6%)	25.0–57.8%	a	8 (25.0%)	12.6–41.7%	a, b
Solar energy	9 (31.0%)	16.6–49.0%	b	8 (27.6%)	14.0–45.4%	a	5 (17.2%)	6.9–33.7%	a, b
Agricultural mechanization	2 (11.8%)	2.5–32.7%	a, b, c	2 (11.8%)	2.5–32.7%	a	4 (23.5%)	8.5–46.7%	a, b
Industrial installations	1 (6.3%)	0.7–25.7%	a, b, c	2 (12.5%)	2.7–34.4%	a	3 (18.8%)	5.6–42.1%	a, b
Carpentry	3 (18.8%)	5.6–42.1%	b, c	5 (31.3%)	13.1–55.6%	a	4 (25.0%)	9.1–49.1%	a, b
Administrative technician	1 (14.3%)	1.6–50.1%	a, b, c	2 (28.6%)	6.5–64.8%	a	1 (14.3%)	1.6–50.1%	a, b
Electronics	3 (50.0%)	16.7–83.3%	b	3 (50.0%)	16.7–83.3%	a	3 (50.0%)	16.7–83.3%	b
# *p*-value	<0.001 *			0.398			0.002 *		
General total	3 (1.4%)	0.4–3.6%		55 (24.9%)	19.5–30.9%		18 (8.1%)	5.1–12.3%	
Technical total	29 (14.7%)	10.3–20.2%		56 (28.4%)	22.5–35.0%		48 (24.4%)	18.8–30.7%	
^ *p*-value	<0.001 *			0.413			<0.001 *		

^ Chi square test. # Fisher’s Exact test. * Significant: *p*-value < 0.05. CI: Confidence interval. HG: Homogenous groups based on post hoc Bonferroni test, homogenous groups had the same symbol “a, b, c”.

## Data Availability

The data sets used and analyzed during the current study are available from the first or corresponding author on reasonable request.
